# Binaural acoustic stimulation in patients with Parkinson’s disease

**DOI:** 10.3389/fneur.2023.1167006

**Published:** 2023-05-05

**Authors:** Alexander Calvano, Lars Timmermann, Philipp Alexander Loehrer, Carina Renate Oehrn, Immo Weber

**Affiliations:** ^1^Department of Neurology, Philipps-University Marburg, Marburg, Germany; ^2^Center for Mind, Brain and Behavior (CMBB), Philipps-University Marburg, Marburg, Germany

**Keywords:** Parkinson’s disease, acoustic stimulation, binaural beats, brainwave entrainment, motor symptoms

## Abstract

Acoustic stimulation can improve motor symptoms in Parkinson’s disease (PD) and might therefore represent a potential non-invasive treatment option. Scalp electroencephalography studies in healthy subjects indicate that specifically binaural beat stimulation (BBS) in the gamma frequency range is associated with synchronized cortical oscillations at 40 Hertz (Hz). Several studies suggest that oscillations in the gamma-frequency range (>30 Hz) serve a prokinetic function in PD. In this double-blind, randomized study, 25 PD patients were recruited. The study was conducted with (ON) and without dopaminergic medication (OFF). Each drug condition consisted of two phases (no stimulation and acoustic stimulation). The acoustic stimulation phase was divided into two blocks including BBS and conventional acoustic stimulation (CAS) as a control condition. For BBS, a modulated frequency of 35 Hz was used (left: 320 Hz; right: 355 Hz) and for CAS 340 Hz on both sides. We assessed effects on motor performance using Movement Disorder Society-Unified Parkinson’s Disease Rating Scale (MDS-UPDRS) and two validated commercially available portable devices (Kinesia ONE™ and Kinesia 360™) measuring motor symptoms such as dyskinesia, bradykinesia, and tremor. Repeated measures ANOVA revealed that BBS improved resting tremor on the side of the more affected limb in the OFF condition, as measured by wearables (*F*_(2,48)_ = 3.61, *p* = 0.035). However, BBS did not exert a general positive effect on motor symptoms as assessed via MDS-UPDRS (*F*_(2,48)_ = 1.00, *p* = 0.327). For CAS, we did not observe an improvement in specific symptoms but rather an overall beneficial effect on motor performance (MDS-UPDRS total score OFF medication: *F*_(2,48)_ = 4.17, *p* = 0.021; wearable scores: *F*_(2,48)_ = 2.46, *p* = 0.097). In this study, we found an improvement of resting tremor when applying BBS in the gamma frequency band OFF medication. Moreover, the positive effects of CAS underline the general positive potential for improvement of motor function by acoustically supported therapeutic approaches. However, more studies are needed to fully characterize the clinical relevance of BBS and to further optimize its ameliorating effects.

## 1. Introduction

Parkinson’s disease (PD) is the second most common neurodegenerative disease after Alzheimer’s disease (1). The clinical presentation of PD is characterized by motor symptoms, such as bradykinesia in combination with rigidity and resting tremor. Growing evidence highlights a key role of altered neural oscillations in the pathology of patients with PD (2). In the healthy brain, frequency bands have been traditionally segregated into delta (0.5–3 Hz), theta (4–7 Hz), alpha (8–12 Hz), beta (13–30 Hz), and gamma (>30 Hz) oscillations and play a key role for information processing (3). In PD, motor symptoms have been especially attributed to enhanced beta and reduced gamma activity in basal ganglia-cortical loops (4).

Currently, symptomatic therapy mainly focuses on dopaminergic agents, that are associated with significant side effects such as dyskinesia and impulse control disorders (5). Increasing motor fluctuations often complicate the oral therapy in later stages of the disease, so that invasive therapy options are to be considered. However, several potential non-invasive therapies have emerged in the recent literature that may be complementary to drug therapy, such as acoustic stimulation. Among others, there are reports that music (6), rhythmic tone sequences (7, 8), and the acoustic presentation of certain frequencies (9) can improve motor symptoms in PD.

Binaural beat stimulation (BBS) represents a specific type of acoustic stimulation and describes acoustic impressions that occur when two sounds with slightly different frequencies are delivered separately to each ear (10). For instance, if a tone with a frequency of 335 Hz is presented to one ear and a tone with 345 Hz to the other ear, a beat signal with a modulated frequency of 10 Hz is produced. The processing of these acoustic impressions presumably takes place in areas of the brain stem and the auditory cortex, resulting in a conscious perception (11, 12). Previous human study results suggest a positive clinical effect of BBS on cognitive functions, such as on creativity (13), working memory (14), and pain (15). In line with this, a recent study by Galvez et al. has found an improvement in working memory performance in PD patients (16). Moreover, BBS has been used as a non-invasive entrainment tool to modulate neural brain activity (17). In this context, BBS is associated with synchronized neural oscillations in the gamma-frequency band (17–19), which are discussed to have prokinetic properties in PD (20, 21). These motor effects in particular await further scientific scrutiny. Here, we assess the effects of BBS in the gamma frequency band on motor symptoms in 25 PD patients and compare them to conventional acoustic stimulation (CAS) and no stimulation.

## 2. Methods

### 2.1. Patients

25 PD patients [10 female, median age (Q1–Q3), 61 (52.25–70)] were recruited from the ward and the outpatient clinic of the Department of Neurology at the University Hospital Marburg (Table 1). The local ethics committee approved the study (study-number: 10/19), which was conducted in accordance with the latest version of the Declaration of Helsinki. Out of all participants, 5 patients were diagnosed with clinically probable PD and 20 patients were diagnosed with clinically established PD according to the Movement Disorder Society diagnostic criteria (22). Enrollment in the study was limited to patients meeting our inclusion criteria, which involved evaluating pre-existing medical conditions, medications affecting auditory and visual perception, as well as inquiring about hearing and vision impairments.

**Table 1:** Demographics and clinical data.

Number of subjects (*n*)	25	
Demographics		
Age (years)	61 (52.25–70)	
Sex (*n*)	Female: 10	Male: 15
Clinical data		
Disease duration (years)	4 (3–9)	
LEDD (mg)	395 (85–725)	
More affected limb (*n*)	Left: 15	Right: 10
Hoehn & Yahr stage	2 (1–2)	
Tremor-dominant PD patients (*n*)	4	
MDS-UPDRS (III)		
OFF No Stim	34 (27–42)	
ON No Stim	22 (13–28)	
Tremor subcores	Left	Right
Postural tremor OFF	0 (0–1)	0 (0–0)
Postural tremor ON	0 (0–0)	0 (0–0)
Action tremor OFF	0 (0–1)	1 (0–1)
Action tremor ON	0 (0–0)	0 (0–0)
Amplitude of resting tremor OFF	0 (0–1)	0 (0–2)
Amplitude of resting tremor ON	0 (0–1)	0 (0–1)

LEDD: Levodopa equivalent daily dose, PD: Parkinson’s disease, MDS-UPDRS: Movement Disorder Society-Unified Parkinson’s Disease Rating Scale, No Stim: no stimulation. Data is presented as median (interquartile range, IQR).

### 2.2. Study design

**Figure 1:**
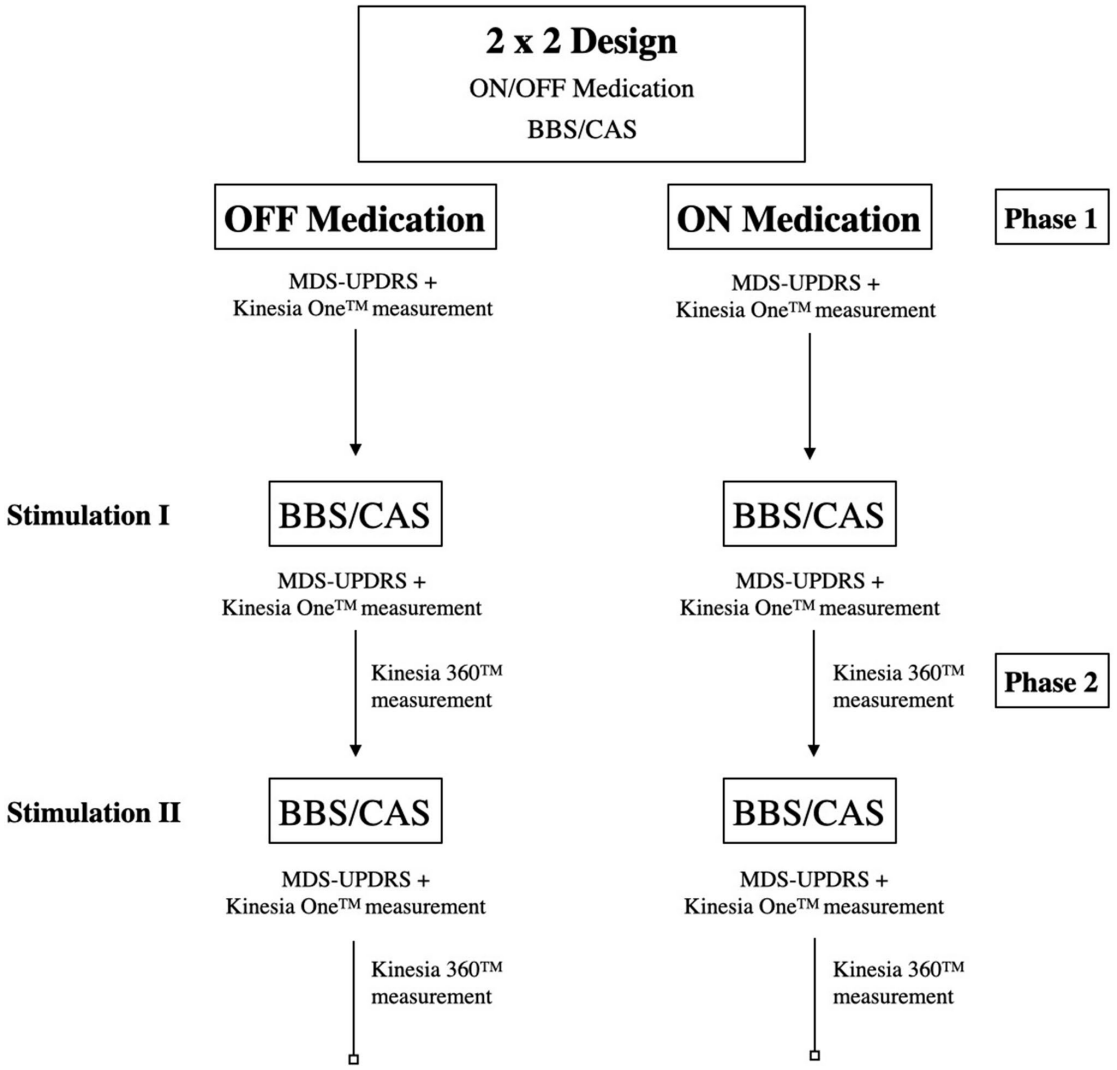
Depiction of the study design. Phase 1: no stimulation, Phase 2: acoustic stimulation. Abbreviations: BBS: binaural beat stimulation, CAS: conventional acoustic stimulation.

This double-blind randomized study was divided into two parts (Figure 1). One part was conducted without dopaminergic medication (OFF condition) and another with the usual medication dose (ON condition). In the OFF condition, patients received medication withdrawal of all dopaminergic agents for at least 12 h prior to the study assessment. In case of treatment with dopamine agonists, medication was discontinued even earlier. The two experimental parts were performed on two separate consecutive days with randomized order during the same time of day. Each experimental part consisted of three stimulation conditions (no stimulation, BBS, CAS). We generated two audio files, one for each stimulation condition (BBS and CAS), each with a length of 30 min (23). Since frequencies in the range of 300 Hz - 600 Hz are best perceived for BBS (24), we used 320 Hz for the left ear and 355 Hz for the right ear. The perceived frequency of BBS was thus at 35 Hz, i.e., in the gamma band (25). Previous studies indicate that 35 Hz corresponds to the highest frequency difference that is not consciously perceived by patients and therefore allows randomization in truly blinded stimulation settings (23, 24). In accordance with the methodology of previous reports that employed a carrier frequency of 340 Hz (13, 26, 27), we presented this frequency on both headphone speakers for the CAS condition. Auditory stimuli were generated using a self-written MATLAB™ script (MathWorks Inc.), utilizing a mathematical sine function. For the verum condition, the left channel was programmed with a frequency of 320 Hz [*f(x) = sin(320)*] and the right channel with a frequency of 355 Hz [*f(x) = sin(355)*]. In the sham condition, we used a control stimulus with a frequency of 340 Hz [*f(x) = sin(340)*] on both the left and right channel. Auditory stimuli were presented at the beginning of *Phase 2*, with either BBS or CAS randomly assigned, and were applied continuously throughout the measurements in *Phase 2*. Following the initial application of the first stimulation setting in *Phase 2*, the alternative stimulation was administered. The acoustic stimuli were presented using a conventional MP3-Player (iPod shuffle, Apple Inc). As BBS can be elicited at very low volumes and sound pressure levels, sometimes even at volumes below the human hearing threshold (28), the volume was set according to the patient’s individual needs and preferences. The two stimulus conditions were renamed in advance to ‘Track 1’ and ‘Track 2’ by an independent clinician, so that neither the patient nor the experimenter knew which condition was tested at the time of measurement. During each of the three stimulation conditions, the experimenter assessed the motor part (Part III) of the standard rating scale of the Movement Disorder Society-Unified Parkinson’s Disease Rating Scale (MDS-UPDRS). The examination was video-recorded and subsequently re-evaluated by an independent clinician. The final MDS-UPDRS scores were taken as the mean of the two evaluations. Subsequently, the Kinesia-ONE™ system, which is a commercially available validated portable device, was used to objectively assess motor performance on the same scale as the MDS-UPRS (0–4) (29–30). In addition to the MDS-UPDRS and wearables scores obtained using standardized tasks, we assessed stimulation effects on walking by using hand and foot sensors of the validated Kinesia 360™ device (31), which were attached to the side of the more affected limb. A table of all subscores is included as Supplementary material. We measured characteristics of gait, such as number of steps during a defined distance (~400 meters) and step length. After completing the study protocol, subjects were asked whether they noticed a difference between the two stimulus conditions, which was consequently documented in the individual case report form (CRF).

### 2.3. Statistical analysis

All statistical analyses were performed using IBM SPSS Version 25 (IBM SPSS Statistics for Mac, Version 25.0. Armonk, NY: IBM Corporation) with an alpha-level of 0.05. We conducted a two-factor repeated measures ANOVA with the dependent variables *MDS-UPDRS, Kinesia ONE™, Kinesia 360™* and independent variable stimulation condition *BBS, CAS, no stimulation*. For the dependent variables, we performed an analysis collapsing bilateral scores, as well as separate analysis of the most affected side. In case of a positive interaction between factors, paired t-tests were conducted as post-hoc tests. Before each analysis, the statistical requirements for the ANOVA were assessed and Greenhouse–Geisser correction was applied in case of unmet sphericity assumption.

## 3. Results

### 3.1. Effects of acoustic stimulation on motor symptoms of both sides

**Figure 2:**
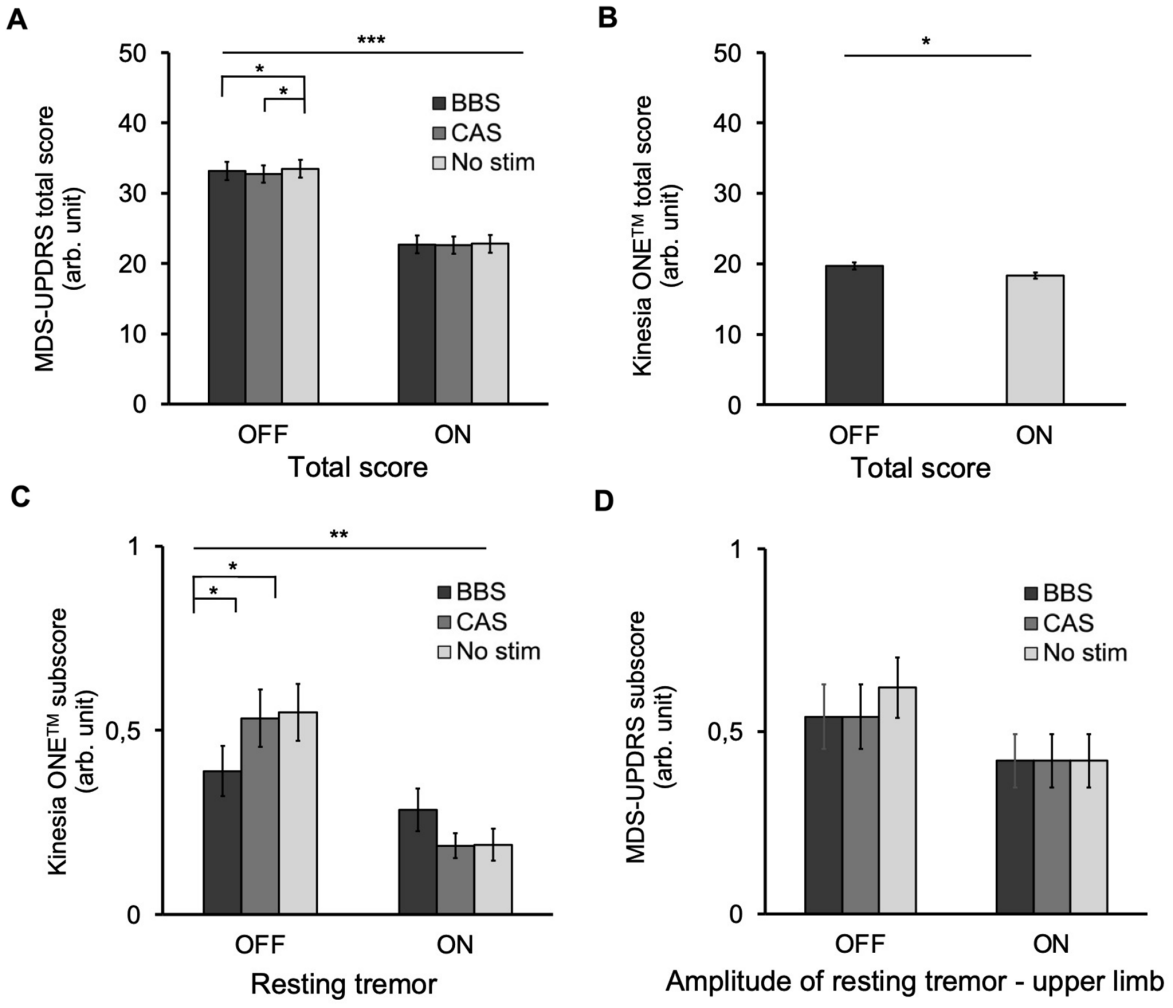
Kinesia ONE™ and MDS-UPDRS total and subscores. **(A)** MDS-UPDRS III total score. Specifically, during OFF medication, BBS and CAS improved motor performance. Furthermore, we observed an effect of medication on MDS-UPDRS III scores. **(B)** Kinesia ONE™ total score. The patients showed significantly less impairment by motor symptoms overall under the influence of medication. **(C)** Kinesia ONE™ subscore: Resting tremor. The participants showed a significantly lower tremor intensity under the influence of medication. In the OFF condition, the Kinesia ONE™ measurement of the side of the more affected limb revealed that patients under BBS had a significantly reduced expression of resting tremor. **(D)** MDS-UPDRS Subscore: Amplitude of resting tremor upper limb. No effects of CAS, BBS or of medication were observed. Error bars represent the standard error of the mean (SEM). ***p < 0.001, **p < 0.01, *p < 0.05 (two-way repeated measures ANOVA, *α* = 0.05).

The repeated-measures ANOVA revealed a main effect of medication (*F*_(1,24)_ = 78.67, *p* < 0.001) and stimulus (*F*_(1.6,39.4)_ = 6.48, *p* = 0.006) on the MDS-UPDRS III total score (Figure 2A). We also found an interaction effect between the two factors (*F*_(2,48)_ = 4.17, *p* = 0.021). Post-hoc analyses showed that both types of acoustic stimulation improved motor symptoms in OFF medication (No Stim vs. BBS: *t*_(24)_ = 2.3, *p* = 0.029; No Stim vs. CAS: t_(24)_ = 3.9, *p* = 0.01), whereas the difference between the clinical effect of the two stimulation conditions failed to reach significance (BBS vs. CAS: *t*_(24)_ = −1.9, *p* = 0.061). For ON medication, there was no improvement of motor symptoms by either acoustic stimulation (No Stim vs. BBS: *t*_(24)_ = 0.8, *p* = 0.43; No Stim vs. CAS: *t*_(24)_ = 1.2, *p* = 0.26; BBS vs. CAS: *t*_(24)_ = −0.9, *p* = 0.38). Considering wearables, we found a main effect of medication (*F*_(1,24)_ = 5.095, *p* = 0.033), but not stimulus (*F*_(1,7,40,3)_ = 1.188, *p* = 0.308) on total Kinesia ONE™ scores and no interaction between factors (Figure 2B, *F*_(2,48)_ = 2.455, *p* = 0.097).

### 3.2. Effects of acoustic stimulation on motor symptoms of the more affected side

However, the analyses of the Kinesia ONE™ data on the side of the more affected limb revealed an effect of the factor medication (*F*_(1,24)_ = 11.360, *p* = 0.003) as well as an interaction (*F*_(2,48)_ = 3.605, *p* = 0.035) between medication and stimulation on resting tremor subscore, as measured by Kinesia ONE™ (Figure 2C). *Post hoc* analyses using paired *t*-tests showed a stimulus effect of BBS (No Stim vs. BBS: *t*_(24)_ = 2.36, *p* = 0.027) but not CAS (No Stim vs. CAS: *t*_(24)_ = 0.15, *p* = 0.882; BBS vs. CAS: *t*_(24)_ = −1.99, *p* = 0.058) for OFF medication, but not ON medication (No Stim vs. BBS: *t*_(24)_ = −1.21, *p* = 0.237; No Stim vs. CAS: *t*_(24)_ = 0.38, *p* = 0.97; BBS vs. CAS: *t*_(24)_ = 1.59, *p* = 0.124). This finding could not be observed in the corresponding MDS-UPDRS score (Figure 2D). In addition, a trend toward a significant interaction for the dyskinesias subscores was found (*F*_(2,48)_ = 2.888, *p* = 0.066).

### 3.3. Effects on walking

Regarding number of steps, Kinesia 360™ results showed no effects for medication (*F*_(1,19)_ = 0.040, *p* = 0.844) or stimulus (*F*_(1,19)_ = 0. 348, *p* = 0.562), and no interaction effect (*F*_(1,19)_ = 0.298, *p* = 0.591).

### 3.4. Acoustic stimulation

No patient reported noticing a difference between the two acoustic stimulation conditions.

## 4. Discussion

The present double-blind study in 25 patients with PD investigates the effects of conventional acoustic stimulation and binaural beat stimulation on motor symptoms in PD in comparison to no acoustic stimulation.

We found that the application of BBS and CAS has a positive effect on motor performance in the OFF medication state as measured by clinical ratings of the MDS-UPDRS III. When looking at individual subscores, the separate analysis of the Kinesia ONE™ data set of the side of the more affected limb shows a significant reduction of resting tremor levels during BBS in the OFF condition. When considering gait parameters, Kinesia 360™ results reveal no effects on number of steps and stride length.

### 4.1. Tremor

Although tremor in PD usually responds well to dopaminergic medication, it can become resistant to pharmacological treatment as the disease progresses and hence remains a therapeutic challenge (32). Before considering advanced treatment options, complementary non-invasive therapies such as auditory stimulation may be employed. To date, however, very few studies have addressed the question of how acoustic interventions can help to alleviate tremor symptoms. Our results indicate a possible improvement in tremor symptoms that was limited to the OFF condition. The overall low motor burden and early stage of disease in our cohort may explain the lack of effect after intake of dopaminergic medication. Of note, the improvement was restricted to the most affected limb, which is similar to the effect of dopaminergic treatment in the early disease where tremor is strongly lateralized (33). In this regard, recent study results suggest that motor burden, on one side, can be correlated to the clinical evaluation for both sides, thus providing a robust prognostic outcome measure (34). Further, these findings are underpinned by recent studies, that described a reduction in tremor levels with the combined use of low-frequency sound and vibrations (20–100 Hz) (35–37). Mosabbir et al. have demonstrated that physioacoustic stimulation with 40 Hz for 12 weeks significantly improved motor symptoms during intervention (37). While these observations suggest a therapeutic effect, the pathomechanisms still remain elusive. Generally, de-or hypersynchronized central networks are discussed as contributors to tremor manifestation, which may be partly regulated by auditory stimulation (38–40). Moreover, low gamma oscillations in the subthalamic nucleus are associated with reduced tremor intensity (41, 42). While Weinberger et al. have shown that this presumably implies a reduced subthalamic gamma activity (41, 42), neural entrainment to gamma frequency BBS has mainly been observed in the temporal cortex (17, 43). On the basis of these findings, it is reasonable to assume that several functional gamma frequency bands are associated with tremor (42). However, the relationship between BBS entrained gamma oscillations and tremor symptoms remains speculative, and further studies should be conducted to determine this association.

### 4.2. Differences in wearable measurements and the MDS-UPDRS III

The MDS-UPDRS (III) is considered the gold standard for the clinical assessment of motor impairments in PD patients (44). Wearable sensors have proven to be feasible in daily life and can complement traditional assessments (45). Our results may suggest a discrepancy in tremor scores as no significant improvement of resting tremor was found in the MDS-UPDRS as opposed to the Kinesia ONE™ measurement. The differences observed between the two methods can mainly be attributed to the high capability of wearables in detecting and measuring subtle changes in motor performance (30). In addition, as MDS-UPDRS scores were calculated as the mean of scores by two independent clinicians, individual differences in these evaluations may also have accounted for this inconsistency. Moreover, since the improvement in resting tremor was modest, the effect may have been subclinical. However, study reports indicate that quantitative mobility measurements can assess features of motor impairments beyond those obtained with the MDS-UPDRS, allowing a more sophisticated characterization of disease heterogeneity (30, 45).

### 4.3. Entrainment

The ability to entrain neural oscillations by BBS is still under debate. Several studies have addressed this question with conflicting results in frequency ranges such as alpha (46), beta (47), theta (48, 49), and gamma (17, 50). Reasons for this include differences in the methodological approach with regard to BBS duration, timing, and applied frequency. For example, while some authors recommend BBS only for short, repetitive intervals (51, 52) as opposed to continuous presentation, a recent meta-analysis by Garcia-Argibay et al. indicates a correlation between the duration of binaural presentation and the extent of effectiveness (53). These observations may contrast with studies that found no corresponding entrainment after both relatively short duration and longer BBS (46, 47, 50). Consequently, further evidence is needed to elucidate the specific contribution of stimulation duration to neural entrainment. Future studies should consider the use electroencephalography (EEG) or magnetencephalography (MEG) measurements to reliably characterize oscillatory activity during BBS. This would provide important information regarding the setting of carrier frequencies, stimulation duration, and frequency of BBS. By gaining corresponding insights, the methodological procedure could be optimized in the future through a uniform approach.

Dyskinesia represents a common side effect of dopaminergic medication (5), possibly due to pulsatile dopamine release (54). Narrowband gamma oscillations (60–90 Hz) appear to be associated with dyskinetic phases (55). In this regard, our results, although not reaching statistical significance, may suggest that gamma frequency BBS could lead to reduced dyskinesia intensity in the ON condition.

To date, there is only one study that has investigated the effects of BBS on PD patients. In three sessions, Galvez et al. assessed the effects of beta BBS (14 Hz) in 14 PD patients on cognitive functions, anxiety, EEG, electrocardiogram, and gait parameters (cadence, step length, speed). Consistent with our findings, no significant improvements in gait characteristics were observed. Interestingly, however, they found a trend of increased cadence, which, in line with previous studies (8, 56), indicates an adaptation of movements to the acoustic signal (16). Underlying mechanisms involve an increased neuronal excitability of spinal motor neurons by reticulospinal pathways, facilitating accelerated initiation of voluntary movements (56). In contrast, we did not apply BBS or CAS in rhythmic beat patterns, as only the effect of the acoustic signal was to be investigated. For BBS, the primary mechanism of action is not targeted towards motor control and movement synchronization, but is rather associated with improvements in cognitive functions (13, 26). Considering the robust relationship between cognition and gait (57), one may assume that BBS may exert an impact on gait by modulating frontal and prefrontal activity underlying dopaminergic and cholinergic substrates (58). However, the efficacy of BBS in ameliorating gait impairments has not yet been established, and further research is needed to explore the potential therapeutic effects and underlying neural mechanisms.

### 4.4. Limitations

Although our study provides interesting insights on the clinical effects of BBS, there are several important limitations. One limitation is reflected in the relative heterogeneity of the patient cohort in regard to Hoehn-Yahr stage, LEDD, symptom severity, and PD subtypes. The inclusion of non-tremor dominant PD patients may have decreased the effect size in our experiment. Based on our findings, future studies might therefore assess the effects of BBS on motor symptoms by exclusively including tremor-dominant PD patients. Previous studies investigating the effects of neural entrainment commonly applied BBS in higher frequency ranges (>40 Hz) (14, 26). Therefore, another limitation is that we were restricted to BBS in the lower gamma frequency band to make the difference in frequencies not perceivable and therefore both stimulation conditions comparable. Moreover, patients were not included if they had hearing or visual impairments that would have hindered the performance of the measurements. However, there was no precautionary testing or standardized verification, leaving the possibility that an unconscious limitation of hearing resulted in a reduced perception of the acoustic application. At last, the majority of results presented in the literature have utilized multiple sessions to assess the effects of BBS on various outcomes (13, 16, 52). Future studies should therefore investigate the potential benefits of employing multiple sessions, which may help to further elucidate the long-term effects of this intervention.

## 5. Conclusion

In summary, this study provides a detailed characterization of the effects of BBS on the motor symptoms in PD with and without medication. We found an improvement of tremor severity in the OFF medication condition when applying BBS in the gamma frequency band compared to CAS and no acoustic stimulation. Thus, these results may open up new avenue of research for non-invasive neuromodulation in PD. However, long-term studies in an at-home setting should be conducted to validate these findings and to determine the clinical significance of BBS.

## Data availability statement

The raw data supporting the conclusions of this article will be made available by the authors, without undue reservation.

## Ethics statement

The studies involving human participants were reviewed and approved by Ethics Committee of the Department of Medicine at the Philipps University of Marburg. The patients/participants provided their written informed consent to participate in this study.

## Author contributions

CO and IW designed the original experiment and developed the data analysis strategy. AC collected the data and analyzed the data under supervision by CO and IW. CO, IW, AC, PL, and LT interpreted the data and drafted the manuscript. All authors contributed to the article and approved the submitted version.

## Funding

PL was supported by the SUCCESS-Program of the Philipps-University of Marburg and the ‘Stiftung zur Foerderung junger Neurowissenschaftler’. CO is funded by the Parkinson Fellowship of the Thiemann Foundation and Junior Principal Investigator award from the Von Behring-Roentgen Foundation. LT reports grants, personal fees, and non-financial support from SAPIENS Steering Brain Stimulation, Medtronic, Boston Scientific, and St. Jude Medical and has received payments from Bayer Healthcare, UCB Schwarz Pharma and Archimedes Pharma and also honoraria as a speaker on symposia sponsored by Teva Pharma, Lundbeck Pharma, Bracco, Gianni PR, Medas Pharma, UCB Schwarz Pharma, Desitin Pharma, Boehringer Ingelheim, GSK, Eumecom, Orion Pharma, Medtronic, Boston Scientific, Cephalon, Abbott, GE Medical, Archimedes, and Bayer. The funders were not involved in the study design, collection, analysis, interpretation of data, the writing of this article, or the decision to submit it for publication. Open Access funding provided by the Open Access Publishing Fund of Philipps-Universität Marburg with support of the Deutsche Forschungsgemeinschaft (DFG, German Research Foundation).

## Conflict of interest

The authors declare that the research was conducted in the absence of any commercial or financial relationships that could be construed as a potential conflict of interest.

## Publisher’s note

All claims expressed in this article are solely those of the authors and do not necessarily represent those of their affiliated organizations, or those of the publisher, the editors and the reviewers. Any product that may be evaluated in this article, or claim that may be made by its manufacturer, is not guaranteed or endorsed by the publisher.
