# Supplementary material for: Binaural acoustic stimulation in patients with Parkinson’s disease
Source: Front Neurol. 2023 May 5;14:1167006. doi: 10.3389/fneur.2023.1167006 (PMC10196363; doi:10.3389/fneur.2023.1167006)
Supplement: Supplementary file 1 [file Table_1.docx]

**Supplementary material**

**Supplementary table**

**Table 1: Subscores of the Kinesia ONE^TM^ and MDS-UPDRS measurements.** Both assessments obtained a score from zero to four, with zero representing no symptoms and four representing severe symptoms. The total score was calculated from the sum of the listed subscores.

| **Measurement** | **Symptom/Body Part** | **Subscore** |
| --- | --- | --- |
| **Kinesia ONE^TM^** | Tremor | - *Resting tremor* - *Action tremor* - *Action tremor* |
|  | Bradykinesia of the upper extremity | - *Finger tapping: Speed* - *Finger tapping: Amplitude* - *Finger tapping: Rhythm* - *Hand movements: Speed* - *Hand movements: Amplitude* - *Hand movements: Rhythm* |
|  | Bradykinesia of the lower limb | - *Toe tapping* - *Leg raising* |
|  | Coordination | - *Alternating movements: Speed* - *Alternating movements: Amplitude* - *Alternating movements: Rhythm* |
|  |  | *Walking* |
|  |  | *Freezing of Gait* |
|  |  | *Dyskinesia* |
| **MDS-UPDRS**  **(III)** |  | *Speech* |
|  |  | *Facial expression* |
|  |  | *Rigidity neck* |
|  |  | *Amplitude of resting tremor lip/jaw* |
|  |  | *Constancy of resting tremor* |
|  |  | *Freezing of Gait* |
|  |  | *Postural stability* |
|  |  | *Posture* |
|  |  | *Global bradykinesia* |
|  | Upper extremity (left and right side respectively) | - *Rigidity* - *Finger tapping* - *Hand movements* - *Pronation-supination* - *Postural tremor hands* - *Action tremor hands* - *Amplitude of resting tremor* |
|  | Lower extremity (left and right side respectively) | - *Rigidity* - *Forefoot tapping* - *Mobility of the legs* - *Standing up from a chair* - *Amplitude of the resting tremor* |
|  |  | *Freezing of Gait* |
|  |  | *Postural stability* |
|  |  | *Posture* |
|  |  | *Global bradykinesia* |
| **Kinesia 360^TM^** |  | *Number of steps* |
